# Predicting Risk of Heat-Related Injuries for Individuals Wearing Personal Protective Equipment Using Smartwatches: Feasibility Observational Study

**DOI:** 10.2196/72324

**Published:** 2025-10-17

**Authors:** Meghan Hegarty-Craver, Donna Womack, Jonathan Thornburg, Timothy Boe, M John Archer, Worth Calfee

**Affiliations:** 1 RTI International, Durham, NC, United States; 2 Office of Research and Development, US Environmental Protection Agency, Research Triangle Park, NC, 27709, United States, 1 9842279699

**Keywords:** wearable, smartwatch, personal protective equipment, PPE, temperature, heat strain, heat-related illness, continuous monitoring

## Abstract

**Background:**

The risk of developing heat-related illness increases when personal protective equipment (PPE) is worn, especially in hot and humid environments. While cooling strategies are effective, they must be applied preemptively or delivered promptly, which can be difficult if individuals are working in dangerous environments or wearing contaminated PPE. Wearable sensors can be leveraged to continuously monitor health including heart rate, respiration rate, blood oxygen levels, and physical activity.

**Objective:**

This study aims to (1) evaluate the use of wearable sensors for monitoring the real-time health of individuals wearing PPE to mitigate the risk of developing a heat-related illness and enable timely intervention, (2) understand how PPE may affect smartwatch data quality and comfort, and (3) identify circumstances in which people wearing PPE may not be able to wear a smartwatch.

**Methods:**

Individuals participating in planned field trainings or exercises where PPE was being worn were asked to wear Garmin Fenix 6 smartwatch (Garmin Ltd) before, during, and after the event to monitor health and recovery. These convenience cohorts were selected to understand the feasibility of using smartwatches with different types of PPE (ie, level C PPE and firefighter gear) for different types of training (ie, a simulated environmental cleanup exercise and skill and tactical maneuver training for new firefighter recruits).

**Results:**

Two data collections were conducted using the Garmin Fenix 6 smartwatch to assess wearability, data quality, and data accuracy. For the first effort, participants wore the watch for 3.9‐5.1 days, and wear compliance ranged from 83.8% to 99.9%. For the second effort, participants wore the watch for the exercise only, which was 3.5 hours. Participants were able to wear the watches for the entire time that they were wearing PPE and did not report any adverse events. Changes in heart rate corresponded with changes in physical activity, providing evidence that physiology can be acceptably monitored during physical activity. Heart rate data artifact ranged between 5.8% and 9.3% and was highest for the control participant (second data collection) who was not wearing PPE.

**Conclusions:**

Based on the results obtained from the 8 pilot users, the Garmin Fenix 6 smartwatch is an appropriate choice for continuously monitoring the health of individuals wearing PPE. The watch can be tolerated for extended wear periods and data quality is sufficient for monitoring heart rate and predicting core body temperature.

## Introduction

### Overview

Heat-related illnesses (HRIs) are common among individuals who must wear personal protective equipment (PPE) to reduce their risk of exposure to chemical, biological, or other threats [1-3]. HRIs can range in severity from heat exhaustion (ie, the individual has an elevated core temperature and symptoms consistent with heat strain) to heat stroke (ie, the individual is no longer able to cool themselves and their temperature climbs dangerously high, causing damage to the central nervous system, which may be fatal) [4]. Heat strain is the body’s physiological response to heat stress (ie, the total heat load from physical exertion, the environment, and clothing impacts) [4]. Symptoms include nausea or loss of appetite, extreme thirst and dehydration, headache, dizziness or confusion, excessive sweating or clammy skin, cramps, fast breathing, and high heart rate (HR) [4].

Wearing PPE while working outdoors or in warm and humid environments increases the risk of developing an HRI [2,3]. Because PPE is designed to reduce exposure to hazards, both heat transfer from the body to the environment (ie, convective heat loss) and cooling through sweating (ie, evaporative heat loss) are disrupted [2]. Core temperature (T_c_) rises as the body is unable to cool itself, and HR and sweat rate continue to increase to try and regain thermal equilibrium [4].

Occupational heat strain can significantly impact productivity, increase risk of injury, and have long-lasting health consequences [1,2]. Professional organizations such as the International Association of Fire Fighters (IAFF), which represents professional firefighters and paramedics across the United States and Canada—and government entities such as the US Environmental Protection Agency (EPA), which is responsible for conducting and supervising cleanup of contamination associated with accidental spills and industrial accidents—are especially interested in strategies to reduce HRI risks imposed by PPE. While cooling strategies are effective [1,2,5], they must be applied preemptively, or treatment must be initiated with enough time to prevent progression of symptoms. This can be difficult if individuals are in hazardous environments (eg, firefighters) or wearing contaminated PPE (eg, workers involved in Hazardous Materials Management [HAZMAT]) [6]. It is likely that a multipronged approach including exposure predictions and individual monitoring is needed to mitigate risk [7].

### Exposure Prediction Models

Several variables affect potential for heat strain injury, including the environment, clothing worn by the individual, physical activity level, and individual physiological factors [7-9]. The Required Sweat Rate model published in 1981 was developed to improve working conditions in industrial settings by removing the subjectivity of perceived heat stress [10]. The Predicted Heat Strain (PHS) Model [8] improved estimations and extended the original model to include predictions of mean skin temperature and sweat rate based on the clothing being worn, as well as rectal temperature and the distribution of heat storage. The US Army Research Institute of Environmental Medicine (USARIEM) Heat Strain Decision Aid (HSDA) is a more recent, spreadsheet-based tool that takes inputs related to the environment, clothing being worn, physical activity level, and physiology to make generalized predictions related to how long it is safe to work under a set of conditions before there is a risk of experiencing thermal strain [9]. This tool is currently used by the US military for establishing safety guidelines.

### Individual Monitoring Using Wearables

#### Overview

Exposure models work well for the constraints under which they were developed (eg, population demographics, activity pattern, type of PPE, and environmental conditions), but accuracy is compromised outside of these bounds [7]. Workers at risk for HRIs are currently instructed how to monitor themselves and colleagues for signs and symptoms, but this is very subjective [3]. The growing availability of commercial off-the-shelf wearable devices and Internet of Things sensors has made it feasible to continuously collect objective health and environmental information. These devices can wirelessly transmit data to cloud services where they can be accessed, processed, and viewed to determine if someone’s health is deteriorating. For robust heat strain monitoring, it is best to continuously measure (vs spot check) several physiological variables such as T_c_, HR, and physical activity level [3,7,11].

#### Core Body Temperature

Monitoring T_c_ is the most direct way of assessing risk for HRI, but accurate and consistent measurements might be difficult to obtain when individuals are wearing PPE [7]. Options for assessing body temperature include esophageal, rectal, tympanic (ie, ear), axillary (ie, armpit), and oral measurements. While esophageal and rectal temperatures most accurately track T_c_, they cannot be measured conveniently [7,12]. Tympanic temperature is accurate to within 1 °C of T_c_ as long as the probe is properly shielded and can be regularly checked, provided that it is safe to temporarily remove PPE [7,12]. Axillary and oral temperatures are the least accurate because it is difficult to properly shield the probe from the environment. These methods of assessment can vary from T_c_ by over 1 °C [12]. Axillary temperature might also be challenging to measure if there are multiple layers of PPE being worn [7].

#### Heart Rate

Sustained high HR can be indicative of overexertion, which increases susceptibility to heat strain injuries [13]. If HR data are updated at a frequency of 1 minute or faster, T_c_ can be estimated using an algorithm such as the one developed by USARIEM [14]. Combined with measures of skin temperature and heat flux, the accuracy of these estimations of T_c_ can be improved [15].

HR can be measured using wearable electrocardiogram patches and chest straps, as well as optical ring- and smartwatch-based sensors. Patches and straps might cause skin irritation, and profuse sweating can cause the adhesive patch to fall off, so they are not appropriate for continuous wear. While optical devices might be better for ambulatory use and long-term monitoring, they typically do not provide the same level of accuracy [16].

#### Activity Level

Measuring activity level allows energy expenditure to be estimated and rest breaks to be tracked. Physical activity level is considered in the HSDA model [9]. Deriving these measurements from 3-axis accelerometer data is more accurate, but most wearables distill this information to either step counts, stair equivalents climbed, or minutes at a given level of movement intensity.

### Considerations for Wearables Pilot Testing

Several wearable sensors can acquire health data relevant to assessing individuals’ risk of developing an HRI. Multisensor systems, especially those that can measure T_c_, HR, and activity level, are preferable.

Systems incorporating electrocardiogram sensors (eg, Zephyr Bioharness 3, Hexoskin smart garments, and VivoSense LifeShirt) are the most accurate for measuring HR. Because close contact with the torso is required for accurate sensing, these platforms are also optimal for measuring respiration rate (RR) and physical activity. In terms of technology adoption, they are limited by the fact that they must be individually sized and can be uncomfortable for long periods of wear (especially when PPE is worn).

The accuracy of systems incorporating optical sensors (eg, smartwatches, armbands, and rings) has improved, and average HR, RR, and activity level can be acceptably measured. Several of these platforms also incorporate measures of blood oxygen saturation (SpO_2_) or skin temperature. Systems with optical sensors are available in more convenient configurations for long-term monitoring of a diverse group of individuals.

After conversations with end users and wearables manufacturers in combination with limited benchtop testing, we selected the Garmin Fenix 6 smartwatch (Garmin Ltd) to pilot test for continuous HRI risk assessment. Other smartwatches (ie, Fitbit Charge 2, Empatica E4 wristband, and the Apple Watch Series 7) and chest straps (ie, Polar H10 and Zephyr Bioharness 3) were reviewed. The Garmin Fenix 6 smartwatch was selected because it can acquire several relevant physiological metrics in a convenient configuration that is appropriate for use with PPE. The Garmin Fenix 6 is ruggedized, operates using button clicks versus a touch screen interface, and has a battery life of 5‐7 days when configured for continuous, high-resolution data acquisition.

Two data collections with convenience cohorts were conducted to determine the feasibility of using the Garmin Fenix 6 smartwatch for monitoring the health of people wearing PPE. The goals of these efforts were to:

1. Determine if a smartwatch is an appropriate configuration for monitoring individuals wearing PPE, including longer-term wear to determine recovery.
2. Determine if the Garmin Fenix 6 can sufficiently monitor vital signs when participants are active doing relevant work; the accuracy of the optical sensors used in smartwatches can be reduced when wrist movement is significant (ie, motion artifact) or for individuals with darker skin tones [16].
3. Gauge the accuracy of the measured and derived health metrics under various test conditions.

## Methods

### Overview

This section describes the study procedure including (1) participant recruitment, (2) study conditions, (3) equipment used for data acquisition, (4) data cleaning and processing strategies, and (5) data analysis. The goal of these data collection efforts was to obtain information related to wear compliance as well as data missingness and accuracy (eg, during activity and rest) for the different types of health data that are available from the Garmin Fenix series smartwatches.

### Study Procedure

The study team identified local training events or exercises where PPE was being worn. The study team selected 2 events or exercises based on the types of activities that were planned, the type of PPE that was being worn, and the availability of study staff and equipment. These 2 events took place in the southeastern United States during the late spring and summer months (Table 1). Individuals who were already participating in these training events were informed by the event organizer of our research effort. In total, 8 individuals older than 18 years were voluntarily recruited from these convenience cohorts. Individuals who had sensitive skin or known allergies to the watch bezel or band material were excluded. The goals of the study, the study protocol, and the associated risks and benefits were verbally reviewed with the volunteers at the location of the training event or exercise prior to participation.

**Table 1.** Two data collections were conducted during field training events or exercises with convenience cohorts.

	Data collection #1	Data collection #2
Participant IDs	EPA101, EPA102, EPA103, EPA104	EPA201, EPA202, EPA203, EPA204, Control
Description	Environmental cleanup training [BT1]	New recruit firefighter training
Duration	4‐5 days	3.5 hours
Environmental conditions	Daytime temperature: 15‐34 °C Daytime humidity: 27%‐100%	Temperature: 30.5‐32.8 °C Humidity: 29%‐38%
PPE^a^	Full-face air purifying respirator, escape mask, hard hat, inner and outer chemical-resistant gloves, and disposable chemical-resistant outer boots	Helmets, gloves, short sleeve cotton T-shirts, protective canvas pants, and boots

a PPE: personal protective equipment.

During the first pilot study, data were collected as part of a multiday environmental field study that required participants to don level C PPE [17]. Participants began wearing the Garmin smartwatch at the start of the field study and continued to wear the watch throughout the field study, including sleep. The participants were instructed to wear the watch snugly on their nondominant hand; the fit of the watch was inspected by study staff at the beginning of the data collection. Long-term monitoring is needed to determine acclimatization status as well as readiness and recovery. The daytime (8 AM-5 PM) temperatures ranged from 15 to 34 °C and the humidity ranged between 27% and 100%.

During the second pilot study, data were collected during a 3.5-hour field training session for newly recruited firefighters. New firefighter recruits are more likely to experience an HRI because they are not acclimated to wearing PPE in warm environments [18]. During the field training session, participants practiced skills and tactical maneuvers such as entering and exiting a structure, carrying and assembling equipment, and climbing ladders. The participants wore typical gear for training outdoors during activities that did not involve extinguishing fires, including helmets, gloves, short-sleeve cotton T-shirts, protective canvas pants, and boots for the duration of the field training session (participants removed their helmets and gloves for a water break occurring approximately halfway through the training exercise). Participants began wearing the Garmin smartwatch on their nondominant hand at the start of the exercise and did not remove it for the duration of the exercise; the fit of the watch was inspected by study staff. Tympanic temperature was taken periodically using a Braun ThermoScan Ear Thermometer (Braun). The temperature for the second pilot study reached 32.7 °C and the humidity was 30%‐35%.

During both data collections, participants were observed, and investigators asked the participants to provide informal feedback related to the smartwatch.

### Data Acquisition

RTI International’s Wearables Research and Analytics Platform (WRAP) [19] was used to obtain high-resolution health data (Table 2) directly from the Garmin Fenix 6 smartwatches using an implementation of the Garmin Companion software development kit. No personally identifiable information was collected or recorded. Participants were assigned a study ID that did not contain personally identifiable information (eg, EPA101) through WRAP. The data were stored and accessed according to this ID and the smartwatch universally unique identifier (UUID). Only the data types described in Table 2 were collected.

**Table 2.** Continuous, high-resolution health metrics were acquired from the Garmin Fenix 6 smartwatch. The data were processed to remove values outside of the valid range before analysis.

	Data update rate	Units	Valid range
Step count	60 s	steps/min	0‐220 [20]
Respiration rate (RR)	60 s	breaths/min	6‐60 [21]
SpO_2^a^_	60 s	percent	60‐100 [22]
Beat-to-beat interval (BBI)	With each heart beat	millisecond	300‐1500 (HR^b^ of 40‐200 beats/min)

a SpO_2_: blood oxygen saturation.

b HR: heart rate.

The WRAP platform includes a mobile app (available on the App Store and Google Play Store) that is capable of 1:1 or 1:many communication between the tablet and wearable(s). Data from the wearable are packaged as JavaScript Object Notation (JSON) files and securely transmitted to a specified end point for storage (eg, the Microsoft Azure endpoint maintained by RTI International). The data can be visualized, and the entire record can be downloaded as a comma-separated value (CSV) file through a browser-based service. WRAP was selected because it can operate in resource-constrained environments as a closed system and does not require connection with Garmin’s cloud services.

### Data Cleaning and Processing

Before processing, artifacts caused by wrist movement and values outside of the expected valid range (Table 2) were removed. This eliminated noise from the incoming signals that can make small changes difficult to detect, which affects model performance.

The cleaned step count and beat-to-beat interval (BBI) data were aggregated into 60-second windows and averaged. The 60-second average BBI was converted to HR and used to estimate core body temperature (eCBT) using the USARIEM algorithm [14]. The 60-second average HR and step count were also used to provide a more granular representation of activity level.

The cleaned step count, RR, SpO_2_, and BBI data were aggregated to 5-minute windows. The average and SD of the 5-minute window were computed for the step count, RR, SpO_2_, and BBI. Time- and frequency-domain heart rate variability (HRV) metrics were calculated using the hrvanalysis package for Python (Aura Healthcare project, Robin Champseix) [23]; outlier and ectopic beats were removed and replaced through linear interpolation.

### Data Analysis

In order to quantify wearability, the total duration of wear and the wear compliance were calculated. An individual was considered to be wearing the watch if, for a given 5-minute window, valid data were present for at least 2 of the 4 metrics described in Table 2.

In order to quantify data quality, the percentage of valid 5-minute windows for all data types was calculated. It should be noted that RR is derived from the same sensor as HR, but SpO_2_ is measured by an independent sensor. The percentage of corrected beats (ie, values outside of the expected range described in Table 2) was also calculated and used to assess HR signal quality. When enough data were present, we assessed if the data quality differed between periods of activity (steps>0) and rest (steps=0).

In order to quantify data accuracy, the recorded and calculated health metrics were compared with expected ranges for active, healthy individuals. In addition, the health metrics were analyzed to determine if they were responsive to activity (steps>0) and rest (steps=0). Where possible, we compared the calculated metrics to ground truth data (eg, eCBT vs tympanic measurement).

### Ethical Considerations

The University of North Carolina Institutional Review Board approved the data collection protocol that was followed in this study (22‐0817) and determined this study to not be human participant research. Study staff obtained verbal consent from the participants at the event or exercise site before data collection began. Study staff explained that participation was voluntary and that individuals could stop participating in the data collection at any time without consequence and that their data would be destroyed. Study staff also explained the risks of the study, including a topical skin reaction to the watch bezel or band material, and informed participants that they could remove the watch at any time if it was causing discomfort or interfering with job duties. No identifying information was collected from participants, and all of the smartwatch data were stored and analyzed using an assigned study identification code (eg, EPA101) that did not contain personally identifiable information. Participants did not receive compensation for volunteering for the study.

## Results

### Overview

This section describes our preliminary findings regarding the (1) wearability, (2) data quality, and (3) accuracy of the Garmin Fenix 6 for continuously monitoring individuals wearing PPE.

### Wearability

Wear compliance is important for assessing acclimatization and recovery regarding wearing PPE in warm and humid environments. In the first data collection, 4 participants (EPA101, EPA102, EPA103, and EPA104) wore the watch for several days, including during a training exercise while wearing PPE. The duration of wear was calculated as the difference between the first and the last valid BBI data point. Wear compliance (ie, percentage of 5-min windows with at least 2 of the 4 smartwatch metrics present over the wear period) was exceptionally high for this cohort, with all 4 individuals wearing the watch for more than 80% of the time (EPA101 wore the watch for 99% of their 3.91 days of participation; EPA102 wore the watch 99.9% of their 5.12 days of participation; EPA103 wore the watch 83.8% of their 4.11 days of participation; EPA104 wore the watch 86.8% of their 4.13 days of participation). This is an important metric to track for long-term monitoring of readiness and recovery.

In the second data collection, 4 different participants (EPA201, EPA202, EPA303, and EPA204) wore the watch for approximately 3.5 hours during a training session. In both pilot studies, the use of PPE did not interfere with data collection, and the Garmin Fenix 6 smartwatch did not impede the protective factor of PPE or negatively impact the safety of the individual.

### Data Quality

In the first data collection, RR and SpO_2_ data were not collected for 2/4 participants due to an error in device initialization. The signal quality was not different between rest and activity for EPA101, but for EPA104, it was somewhat degraded during activity (Table 3). For EPA104, there are fewer valid windows for RR and SpO_2_ data during activity (>0 steps over a 5-minute window) compared to rest (0 steps over a 5-minute window); RR and SpO_2_ data quality were comparable between activity and rest for EPA101. HR and HRV data quality were not impacted by activity for either participant.

**Table 3.** Data quality was compared for 2 participants in the first data collection with valid heart rate, HRV^a^, RR^b^, and SpO_2_^c^ data; a device initialization error resulted in missing respiration rate and SpO_2_ data from the other 2 participants.

Study ID	EPA101		EPA104	
Physiological state	Rest (%)	Active (%)	Rest (%)	Active (%)
% Correction	8.7	8.9	6.6	6.4
% Valid HRV windows	98	100	100	97
% Valid RR windows	97	98	99	85
% Valid SpO_2_ windows	93	94	97	89

a HRV: heart rate variability.

b RR: respiration rate.

c SpO_2_: blood oxygen saturation.

The second data collection included 1 participant (EPA201) who had darker skin [16]; this participant had degraded SpO_2_ signal quality compared to the other participants as indicated by the reduction in valid SpO_2_ values for EPA201 (Table 4). The second data collection also included 1 participant (Control) who was not active (ie, average step count of 4.5 steps/min vs 16.1 steps/min for the other participants) and was not wearing PPE, but who was exposed to the same environmental conditions. This participant had reduced HR signal quality compared to the other participants (Table 4), which could be attributed to the comparatively looser fit of their watch (this participant was female and had a smaller wrist compared to all of the other participants who were male).

**Table 4.** Data quality was compared for 5 participants in the second data collection.

Study ID	Control (%)	EPA201 (%)	EPA202 (%)	EPA203 (%)	EPA204 (%)
% Correction	9.3	6.3	6.8	5.8	7.4
% Valid HRV^a^ windows	100	100	100	100	100
% Valid RR^b^ windows	100	97	97	97	95
% Valid SpO_2_^c^ windows	100	74	97	100	97

a HRV: heart rate variability.

b RR: respiration rate.

c SpO_2_: blood oxygen saturation.

### Data Accuracy

**Figure 1.**
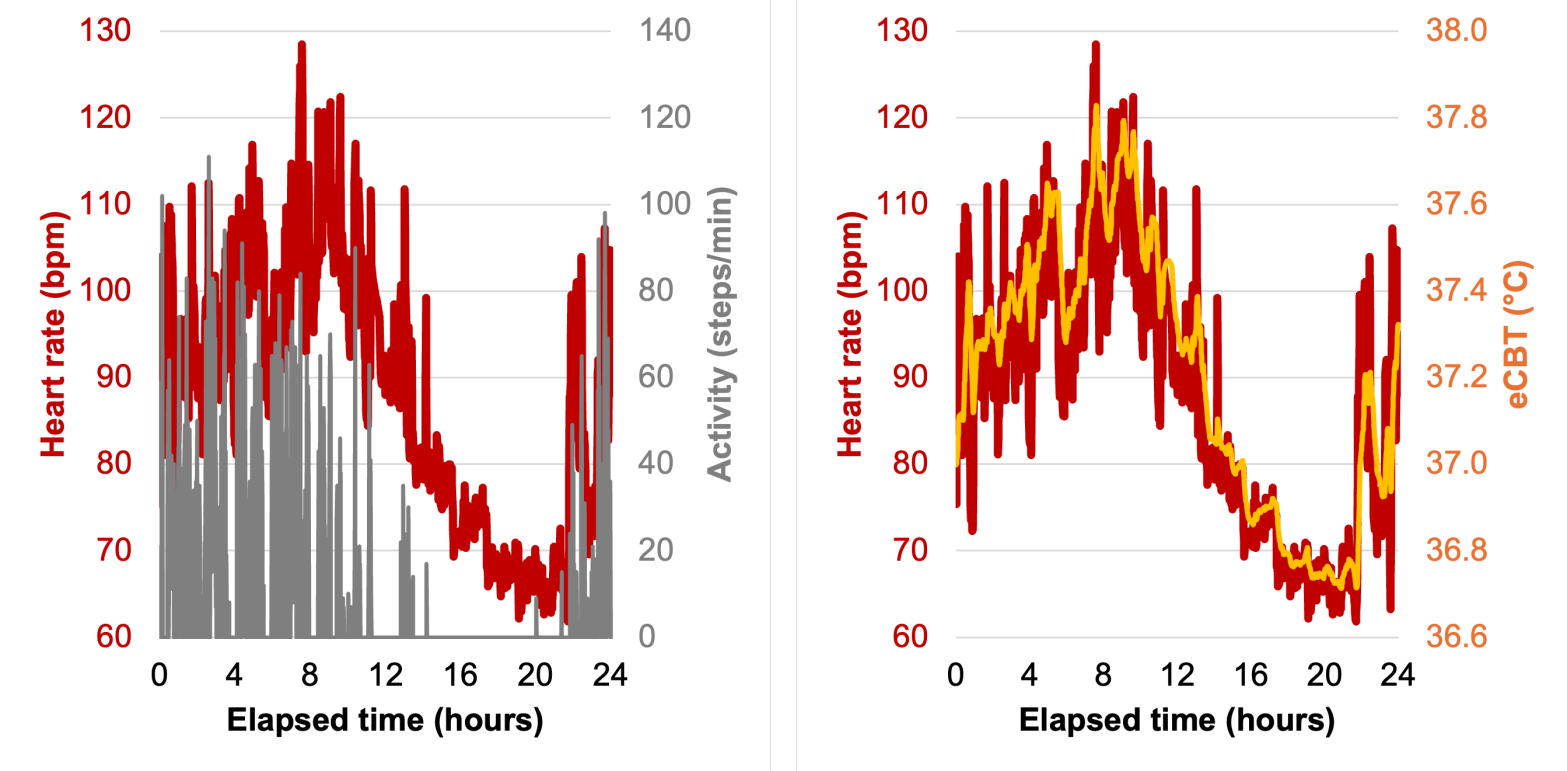
Example activity, heart rate, and estimated core body temperature data for a participant in the first multiday data collection demonstrate that the heart rate and estimated core body temperature measured using the Garmin Fenix 6 increase with physical activity and rest. bpm: beats per minute; eCBT: estimated core body temperature.

All measured and estimated metrics (Tables S1 and S2 in Multimedia Appendix 1) were within physiologically acceptable ranges (Table 2) for active, healthy individuals. For the first data collection, HR and eCBT increased with activity and decreased during rest as expected (core temperature was initialized to 37 °C for all individuals; Figure 1), but RR and SpO_2_ did not (Table S1 in Multimedia Appendix 1).

**Figure 2.**
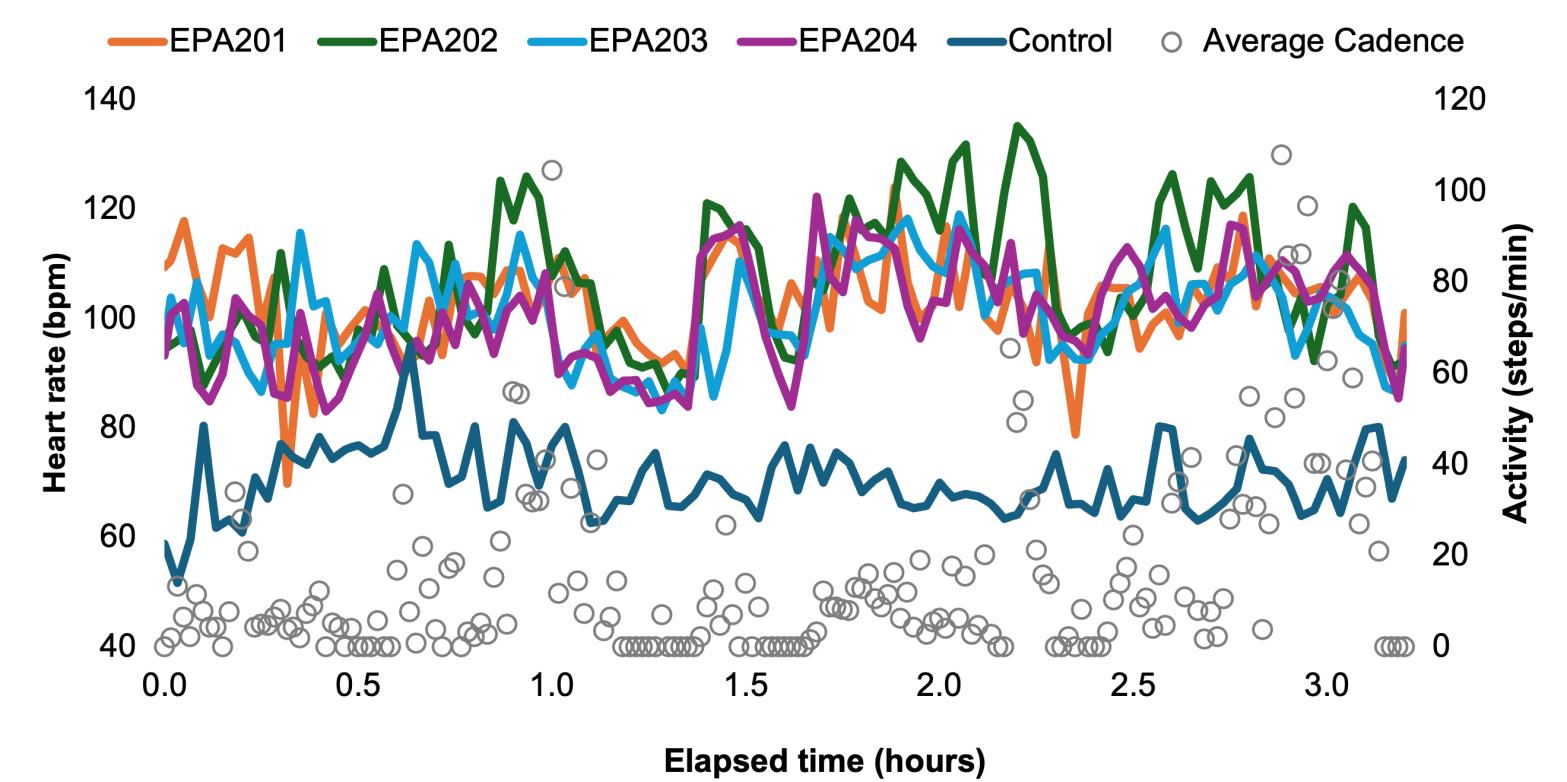
Heart rate comparisons for 4 active participants and 1 control participant (who was exposed to the same environmental conditions) during the second 3.5-hour data collection demonstrate that the Garmin Fenix 6 smartwatch can be used to measure heart rate differences during physical activity. bpm: beats per minute.

**Figure 3.**
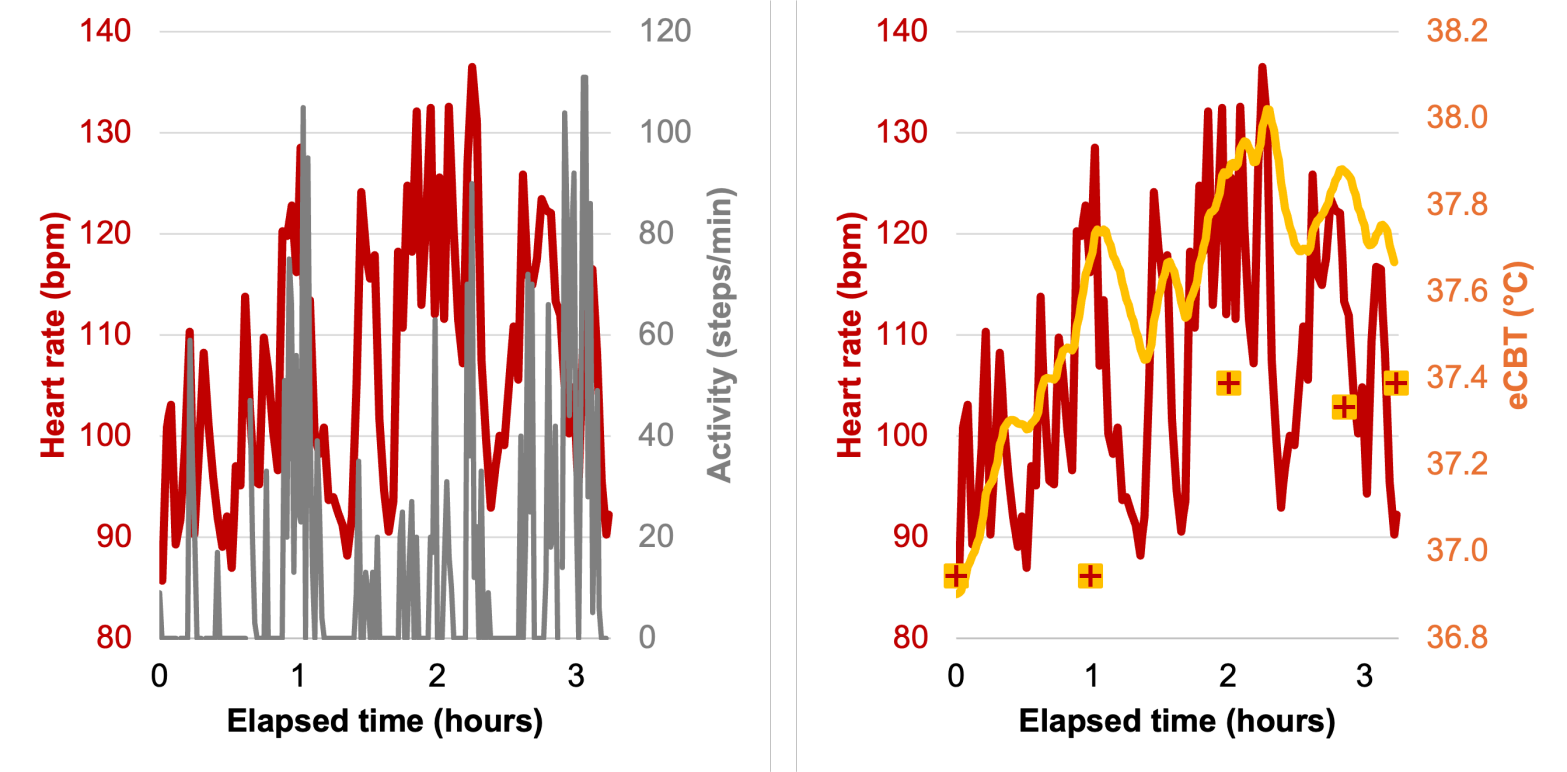
The heart rate measurements (shown as a dark red line) track with physical activity (shown as a gray line), but the core body temperature that is estimated from heart rate (shown as a gold line) overestimates the tympanic temperature (shown as gold square markers). bpm: beats per minute; eCBT: estimated core body temperature.

In the second data collection, there was a difference in HR, eCBT, and RR between the 1 control and the 4 active participants (Figure 2; Table S2 in Multimedia Appendix 1). For this study, the T_c_ used in the USARIEM algorithm was initialized to the first measured temperature (using a Braun ThermoScan Ear Thermometer). The estimated (via the USARIEM algorithm) and measured temperatures deviated after this initial measurement and were overestimated on average by 0.5 °C for the active participants and underestimated for the inactive participant by 0.4 °C (Figure 3; Table S3 in Multimedia Appendix 1).

## Discussion

### Overview

Continuously monitoring individuals using wearables can provide near-real-time health information. Vital sign measurements such as HR, RR, SpO_2_, and temperature are available on many commercial off-the-shelf wearables. Providing this information to workers and the personnel responsible for ensuring their health can be used to better inform current workload and recovery. This is critical for promptly initiating cooling strategies, which can be difficult if individuals are working in dangerous environments or wearing contaminated PPE.

### Principal Results

Both data collection efforts confirmed that the use of the Garmin Fenix 6 smartwatch for continuous health assessment was feasible. Feedback was informally collected for the 8 participants who wore the smartwatch while wearing PPE. Based on this feedback and observations from study staff, the Fenix 6 smartwatch did not interfere with work while wearing PPE. In addition, results from the first data collection showed that the smartwatch could be worn continuously for several days (average of 93% wear compliance over 4.3 days for the first pilot study). The participants did not find the watch to be uncomfortable, and one individual noted that it was about the same size as the watch they regularly wore. However, the Fenix 6 was larger and bulkier than the other smartwatches and activity trackers worn by most of the test participants (eg, the Apple Watch and various Fitbit models), but all noted that the battery life of the Fenix 6 was superior.

In addition, the data quality from the Garmin Fenix 6 sensors was sufficient to report 60-second and 5-minute BBI, HRV, and RR metrics and was not significantly affected by skin tone or arm and hand movement. These metrics also showed appropriate changes with activity level, although data quality was degraded during activity in some cases. SpO_2_ was reported less often during activity, especially for the individual who had a darker skin tone.

Core body temperature was estimated from 60-second average HR measurements using the USARIEM algorithm [14]. The original algorithm was developed using HR data from a Polar chest strap which, if worn correctly, is more accurate than HR data from a smartwatch [16]. While eCBT was within the expected range, the estimated and measured body temperatures disagreed and were overestimated on average by 0.5 °C for the active participants and underestimated for the inactive participants by 0.4 °C. Buller et al [14] noted that core body temperature would be underestimated at low workloads, which is consistent with the results for the control participant in the second pilot study.

### Impact

The goal of this work is to consider the use of wearable sensors to provide actionable information related to the risk of developing an HRI. Our vision is to continuously monitor individuals to mitigate adverse health events in the field as well as track the recovery phase to determine overall readiness to work. This information could be used to initiate timely intervention versus relying on subjective symptom monitoring. In addition, this information could feed exposure prediction models like the HSDA to either provide more accurate information for initial predictions or update forecasts during the workday.

**Figure 4.**
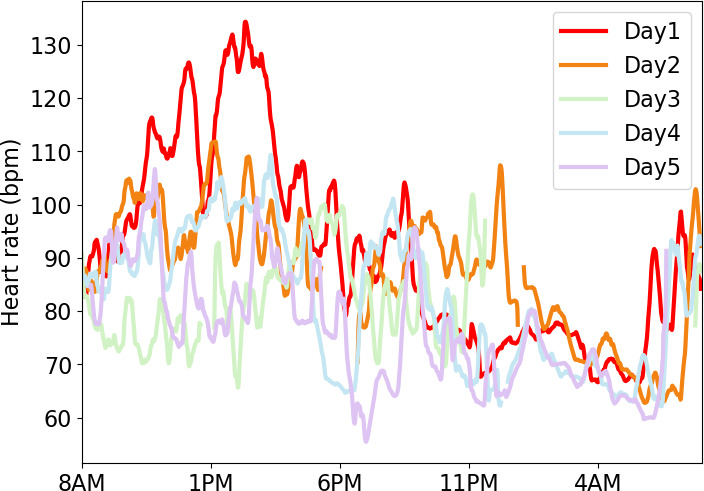
Day 1 (shown as a bright red line) was EPA104’s most active day and their heart rate reached its highest level, exceeding 125 bpm for close to 2 hours, and recovered during the evening hours. bpm: beats per minute.

**Figure 5.**
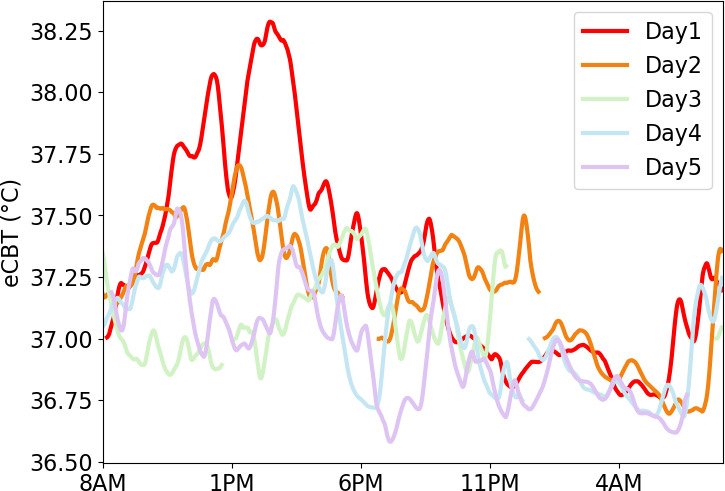
Core body temperature was estimated from the average heart rate over 5 days and nights for participant EPA104 and reached its highest level of 38.3 °C on day 1 which was the participant’s most active day. eCBT: estimated core body temperature.

As an example, during the first pilot test, participant EPA104 attained a maximum HR of over 140 bpm and eCBT of 38.3 °C on the first day of testing. This individual maintained an HR of 125 bpm or higher for 1.8 hours on that day, which is consistent with moderate exercise. When examining their recovery trajectory over the measurement period of 5 days and nights, their HR (Figure 4) and eCBT (Figure 5) were elevated in the earlier part of sleep on the second night following another active day. This might indicate that they did not fully recover from this sustained exercise.

### Comparison with Prior Work

Several recent studies have assessed the prevalence of HRIs among different groups of individuals who wear PPE or work outdoors [1-3,6,24]. The majority of these studies use survey-based tools to collect demographic information, characteristics of the workplace, the prevalence of heat strain symptoms, and accessibility to mitigation strategies (including education). The common main finding from these studies is that the prevalence of HRIs is underreported because people might ignore chronic, low-grade symptoms [3]. Wearables can provide visibility into health metrics that are sensitive to HRI and quantify the effects of these subacute events.

Runkle et al [24] outfitted a small group of workers with a wearable temperature sensor that measured the ambient environment (Thermochron iButton; iButtonLink) and a Garmin Vivoactive smartwatch. They measured HR at a 60-second resolution and postprocessed data to look for periods of sustained high HR (ie, 5-minute average at or exceeding 180 bpm). Our work extended Runkle et al [24] by including additional metrics that are relevant to monitoring for HRI. Studies have been dedicated to generating data to develop and validate models that estimate T_c_ from HR and skin temperature [13-15]. Our work extended data collection from HR straps to a more convenient configuration for long-term wear (ie, smartwatch).

### Limitations of This Study

Participants were drawn from a pool of volunteers who were taking part in ongoing training events or exercises involving the use of PPE. While convenience samples are representative of the target end users, the convenience samples from our pilot studies were not diverse. Across both groups, only males volunteered (the female control participant was not wearing PPE and was not engaged in the training events or exercise). Sex-based differences are known in the relationship between HR and core body temperature, as well as physiological responses to exercise and dehydration [25,26]. In addition, 7 of the 8 users were Caucasian, and there are known differences in the accuracy of optical sensors based on skin tone [16].

To assess feasibility, participants would ideally wear the watch for at least several days. Logistics around remote sensor distribution and collection hindered data collection outside of the scheduled training period. For both data collections, participants began wearing the watch at the start of the exercise. For the first data collection, study staff remained onsite for several days as part of other activities, enabling watches to be worn for an extended period of time. In the second data collection, study staff could not stay on site, and watches were returned immediately following the exercise. In addition, this study would benefit from a structured feedback survey to obtain information about smartwatch use with PPE versus the informal methods that were used.

Accuracy assessments should be made by comparing the data obtained from wearables to “gold standard” sensors. These sensors are typically used in clinics and are not appropriate for continuous monitoring in the field. In the second data collection, an ear thermometer was used to assess body temperature quickly and conveniently, but tympanic temperature varies more than core temperature [27], so other options should be evaluated.

### Recommendations

The pilot studies demonstrated the feasibility of using the Garmin Fenix 6 smartwatch to continuously monitor the health of individuals working in the field while wearing PPE. For future data collection efforts, we recommend:

1. Intentionally recruiting a more diverse group of participants in order to ensure that the results can be translated to all potential end users. For example, we found that the SpO_2_ readings were less reliable for individuals with darker skin tones when they were active. This means that models should not rely on this measurement for active individuals and further testing is needed to determine validity at rest.
2. Including higher levels of PPE and a greater diversity of tasks in the data collection period.
3. Asking participants to wear the smartwatch for a longer period of time in order to capture data before and after the exercise. Baseline data could be used to explain between-person differences in response to activity and heat exposure. Recovery data could be used to understand what data are needed to accurately predict the risk of developing heat strain over days of repeated exposure.
4. Exploring other sensors to acquire more accurate information during training (eg, a sensor that measures core temperature versus estimating it from HR) and verify smartwatch measurements.
5. Asking participants to keep an activity log and track fluid intake to verify the smartwatch measurements and better understand periods when the smartwatch data diverge from more accurate sources.
6. Using a structured feedback survey to collect information about the smartwatch and monitoring technology.

### Conclusions

Occupational heat strain can significantly impact productivity, increase the risk of injury, and have long-lasting health consequences. Wearables can be used to continuously monitor health. We conducted 2 data collections to determine the feasibility of using wearables to monitor for risk of HRI. Results were favorable, and we are planning a larger-scale study involving a more diverse group of participants, longer data collection periods, and additional, higher-resolution sensors.

## Supplementary material

10.2196/72324 Multimedia Appendix 1 The supplementary tables containing detailed physiological information for the 8 test participants.

## Abbreviations

BBI: beat-to-beat interval
bpm: beats per minute
CSV: comma-separated value
eCBT: estimated core body temperature
EPA: US Environmental Protection Agency
HAZMAT: Hazardous Materials Management
HR: heart rate
HRI: heat-related injury
HRV: heart rate variability
HSDA: Heat Strain Decision Aid
IAFF: International Association of Fire Fighters
JSON: JavaScript object notation
PHS: Predicted Heat Strain
PPE: personal protective equipment
RR: respiration rate
SpO_2_: blood oxygen saturation
T_c_: core temperature
USARIEM: US Army Research Institute of Environmental Medicine
UUID: universally unique identifier
WRAP: Wearables and Analytics Platform
