# Supplementary material for: Predicting Risk of Heat-Related Injuries for Individuals Wearing Personal Protective Equipment Using Smartwatches: Feasibility Observational Study
Source: JMIR Form Res. 2025 Oct 17;9:e72324. doi: 10.2196/72324 (PMC12533933; doi:10.2196/72324)
Supplement: Multimedia Appendix 1 [file formative-v9-e72324-s001.docx]

## Supplemental Materials

**Table S1.** All available health metrics for the first data collect are provided in Table S1. There was a problem with device initialization for participants EPA102 and EPA103 and only HR data was collected for these participants.

| **Study ID** | EPA101 | EPA102 | EPA103 | EPA104 |
| --- | --- | --- | --- | --- |
|  |  |  |  |  |
| **Activity (steps/min)** |  |  |  |  |
|  | 0 (rest) | -- | -- | 0 (rest) |
|  | 32.0 (active) | -- | -- | 35.4 (active) |
| **HR (bpm)** |  |  |  |  |
|  | 83.7 (rest) | 74.7 (all) | 76.7 (all) | 82.3 (rest) |
|  | 99.6 (active) |  |  | 99.2 (active) |
| **eCBT (^o^C)** |  |  |  |  |
|  | 37.1 (rest) | 36.9 (all) | 37.0 (all) | 37.1 (rest) |
|  | 37.4 (active) |  |  | 37.4 (active) |
| **RR (breaths/min)** |  |  |  |  |
|  | 13.9 (rest) | -- | -- | 14.5 (rest) |
|  | 13.1 (active) |  |  | 14.0 (active) |
| **SpO_2_** |  |  |  |  |
|  | 94.3 (rest) | -- | -- | 93.6 (rest) |
|  | 94.9 (active) |  |  | 94.3 (active) |

**Table S2.** All available health metrics for the second data collect are provided in Table S2. Comparison of health metrics across individuals may be used to assess the effects of differences in workload and acclimatization on physiology. The health metrics are similar between the four test participants (EPA201-204). The Control participant was not as active (lower step count) as the four other participants.

| **Study ID** | Control | EPA201 | EPA202 | EPA203 | EPA204 |
| --- | --- | --- | --- | --- | --- |
|  |  |  |  |  |  |
| **Activity (steps/min)** |  |  |  |  |  |
|  | 4.5 (avg) | 15.7 (avg) | 15.1 (avg) | 15.8 (avg) | 18.2 (avg) |
| **HR (bpm)** |  |  |  |  |  |
|  | 71.0 (avg) | 102.9 (avg) | 107.1 (avg) | 100.8 (avg) | 100.5 (avg) |
|  | 97.5 (max) | 124.1 (max) | 136.5 (max) | 119.1 (max) | 122.4 (max) |
| **eCBT (^o^C)** |  |  |  |  |  |
|  | 36.9 (avg) | 37.5 (avg) | 37.6 (avg) | 37.5 (avg) | 37.4 (avg) |
|  | 37.3 (max) | 37.7 (max) | 38.0 (max) | 37.7 (max) | 37.7 (max) |
| **RR (breaths/min)** |  |  |  |  |  |
|  | 11.6 (avg) | 14.2 (avg) | 14.0 (avg) | 13.7 (avg) | 13.9 (avg) |
|  | 15.0 (max) | 16.0 (max) | 16.0 (max) | 16.0 (max) | 16.0 (max) |
| **SpO_2_** |  |  |  |  |  |
|  | 94.3 (avg) | 95.8 (avg) | 97.8 (avg) | 95.7 (avg) | 95.0 (avg) |
|  | 93.0 (min) | 92.0 (min) | 95.0 (min) | 94.0 (min) | 94.0 (max) |

**Table S3.** During the second data collect, tympanic temperature was measured at 4 different time points. The difference between the estimated and measured tympanic temperature indicates consistent overestimation for the active participants and underestimation for the Control. The initial estimate was set to the measured temperature.

| **Study ID** | Control | EPA201 | EPA202 | EPA203 | EPA204 |
| --- | --- | --- | --- | --- | --- |
|  |  |  |  |  |  |
| **Initial Temp (^o^C)** |  |  |  |  |  |
|  | 37.3 | 36.9 | 36.9 | 37.0 | 36.6 |
| **Timepoint 1 (ΔT ^o^C)** |  |  |  |  |  |
|  | -0.3 | 0.6 | 0.7 | 0.4 | 0.9 |
| **Timepoint 2 (ΔT ^o^C)** |  |  |  |  |  |
|  | -0.4 | 0.8 | 0.5 | 0.6 | 0.8 |
| **Timepoint 3 (ΔT ^o^C)** |  |  |  |  |  |
|  | -0.5 | 0.4 | 0.5 | 0.4 | 0.8 |
| **Timepoint 4** **(ΔT ^o^C)** |  |  |  |  |  |
|  | -- | 0.3 | 0.3 | 0.2 | 0.5 |
